# The Role of Rumination and Negative Affect in Meaning Making Following Stressful Experiences in a Japanese Sample

**DOI:** 10.3389/fpsyg.2018.02404

**Published:** 2018-11-28

**Authors:** Namiko Kamijo, Shintaro Yukawa

**Affiliations:** ^1^Graduate School of Comprehensive Human Sciences, University of Tsukuba, Tsukuba, Japan; ^2^Faculty of Human Sciences, University of Tsukuba, Tsukuba, Japan

**Keywords:** meaning making, intrusive rumination, deliberate rumination, threat, negative affect, regret

## Abstract

Stressful experiences can lead to meaning making that is seen as central in adjustment. Although rumination and negative affect are important factors of meaning making, little is known about the mechanisms involved. This study aimed to examine the meaning making process, focusing on the role of intrusive and deliberate rumination and negative affect. The principal hypotheses were as follows: negative affect is positively related to threat evaluation and intrusive rumination, while regret and guilt are positively related to deliberate rumination; intrusive rumination is negatively related to finding meaning, whereas deliberate rumination is positively related to finding meaning. A total of 383 undergraduate students were asked to remember their most stressful life event and complete a questionnaire containing the Event Related Rumination Inventory and items about negative affect, threat evaluation, and finding meaning about the stressful life event. For 342 of the final sample, structural equation modeling based on the study hypotheses showed that both deliberate and intrusive rumination immediately after the event were positively associated with finding meaning. Intrusive rumination at present, however, was negatively related to finding meaning. This study also revealed the effects of negative affect: helplessness, sadness, and fear induced intrusive rumination; moreover, regret was positively associated with deliberate rumination.

## Introduction

### Stressful Life Events and Meaning Making

Many individuals face highly stressful and traumatic experiences (e.g., bereavement, natural disaster, murder, traffic accident, divorce), which typically lead to serious posttraumatic symptoms; however, many people are able to understand and interpret their individual experience in their own way. Such cognitive coping is often referred to as meaning making (Park, 2010), which may play a role in adaptation to such experiences. Meaning making was found to be central in recovering from stressful experiences such as bereavement, illness, and terrorist attacks (Gillies and Neimeyer, 2006; Kernan and Lepore, 2009; Park et al., 2012). It alleviates posttraumatic symptoms and brings about positive changes following the experience (Bower et al., 1998; Tolstikova et al., 2005; Updegraff et al., 2008).

Park (2010) proposed the meaning making model, which is a useful theoretical framework for understanding the processes of meaning making. This model identifies two levels of meaning: global and situational. Global meaning refers to individuals’ general orienting systems, consisting of beliefs, goals, and worldview. Individuals’ deeply ingrained global meaning involves the belief that the world is benevolent, predictable, and meaningful, and the self is worthy (Janoff-Bulman, 1989), and life is lived based on these upheld beliefs. On the other hand, situational meaning consists of appraisals of a specific situation. The model posits that when individuals perceive a discrepancy between their global and situational meanings, they experience distress, which leads them to meaning-making efforts to mitigate the discrepancy.

Because this model represents restrictive processes of meaning making, it is insufficient to elucidate the cognitive processes that take place after being motivated to make meaning. Furthermore, not all individuals who experience stressful and traumatic events report having any answers to questions such as “Why did this event happen?” or “Why me?” Moreover, chronic meaning-making efforts may even enhance their distress (Updegraff et al., 2008; Davis and Novoa, 2013). However, few studies have examined the factors that contribute to chronic meaning-making efforts from the viewpoint of meaning-making processes. It is thus necessary to clarify the components of meaning-making processes to resolve the problems mentioned above. Therefore, we first focused on rumination as a critical factor in meaning making.

### Two Types of Rumination

According to Greenberg (1995) and Park and George (2013), rumination concerning stressful experiences is an important factor in meaning making because it promotes trauma reappraisal or schematic revision. Moreover, Tedeschi and Calhoun (2004) proposed two types of rumination identified in the cognitive processing of meaning making, which is a necessary step in adjustment and positive change. One type is intrusive rumination, which involves unintentional and unwanted thinking and images that are difficult to control, with contents related to the stressful events. The discrepancy between global and situational meanings leads to intrusive rumination (Greenberg, 1995; Park, 2008). Intrusive rumination is accompanied by substantial emotional distress and negative affect (Roberts et al., 2006; Updegraff et al., 2008; Kernan and Lepore, 2009) but decreases when individuals find some kind of meaning in their experience (Silver et al., 1983). The other type of rumination is deliberate rumination, which involves voluntarily and purposely trying to understand events and their implications (Calhoun et al., 2000), for example, answering questions such as “Have I learned anything?” or “Has the experience changed my beliefs about the world?” Deliberate rumination is more likely to be related to posttraumatic growth (PTG), in which positive psychological changes result from the struggle with a highly stressful life event, whereas intrusive rumination, which is not controlled by the individual, is more likely to be related to various kinds of posttraumatic stress. Consequently, because intrusive rumination leads individuals who experience stressful events to focus on negative aspects of their experience, they may have difficulty in finding meaning, with the time spent searching for meaning being prolonged, subsequently increasing their distress. On the other hand, because deliberate rumination may shed light on multilateral and positive aspects of their experience, this type of rumination may promote understanding and help to find meaning, value, and significance in the experience.

However, intrusive rumination is also necessary to trigger the cognitive processes toward positive change (Taku et al., 2009; Wu et al., 2015), and is a normal response that immediately follows stressful and traumatic experiences (Joseph et al., 2012). Intrusive rumination is also a coping mechanism that is important as a survival strategy to hedge from a menace quickly. Thus, intrusive rumination may be as important as deliberate rumination in order to find meaning. In previous research, the role of these ruminations types has not been studied in depth and, in fact, no previous studies on associations between the two types of rumination and meaning making exist in the literature. Furthermore, it is possible that a chronic intrusive rumination leads to post-traumatic stress disorder (PTSD) or increased stress response (Greenberg, 1995; Calhoun et al., 2000; Michael et al., 2007). Specially, intrusive rumination may have a different function according to its time of occurrence. In previous studies, intrusive rumination immediately after the stressful event was not related to poor mental health, but intrusive rumination at present (at the point of research) was positively associated with stress responses (Taku et al., 2008, 2009; Nightingale et al., 2010). In consideration of these results and the function of intrusive rumination, although such kind of rumination occurring at the time of the experience may reflect the stress response at the time, its effect on finding meaning may be very small. However, high levels of intrusive rumination after a certain time may lead individuals to focus on negative aspects of the stressful event, and it is assumed that they might continue searching for meaning unsuccessfully or generate negative meaning. Moreover, if intrusive rumination immediately after the event is a normal response and predictor of getting over the experience, intrusive rumination could promote deliberate rumination. Thus, research needs to focus on the timing of rumination and meaning making processes with the ultimate goal of clarifying the factors that promote or inhibit finding meaning.

It is assumed that a factor influencing the frequency of rumination is the subjective threat evaluation of the stressful and traumatic experience. Park (2010) proposed that subjective threat evaluation may be used as an index of the degree of discrepancy between global and situational meanings. The perception of threat to the self or worldview may motivate individuals to review and revise their values and priorities, which are part of their global meaning (Davis and Macdonald, 2004). Based on their studies, threat evaluation of the stressful experiences is an important factor for motivation to find meaning, which involves revising one’s own worldview and common sense, or changing the interpretation of the experience. Given that the discrepancy between global and situational meanings induces intrusive and deliberate rumination (Greenberg, 1995; Park, 2008; Taku et al., 2008), it is assumed that highly threatening experiences promote both intrusive and deliberate rumination in order to concurrently find meaning.

Therefore, this study examined the meaning making process to clarify the factors promoting and inhibiting finding meaning. Simultaneously, we examined the relationship between finding meaning and the frequency and timing of the two types of rumination and between these and threat evaluation.

### Negative Affect in Meaning Making and Rumination

When individuals go through stressful and traumatic life events, they experience strong emotions. George and Park (2013) focused on the influence of negative affect on meaning making following stressful and traumatic experiences. Meaning making was found to mitigate not only depression and PTSD but also negative affect (Park, 2010; George and Park, 2013). Most previous studies showed that finding meaning reduced the degree of negative affect when experiencing stressful events (Park, 2010; George and Park, 2013). However, no precise has been identified relationship between the various types of negative affect immediately after a stressful event and meaning making, as researchers tend to handle negative affect as a general construct. Given that there are many different kinds of negative affect as described below, each one of them may have distinctive effects on meaning making. Hence, elucidation of the negative affect roles in the meaning-making process, in particular the relationships between negative affect and the two types of rumination may contribute to increasing our knowledge to prevent the prolongation of meaning-making efforts and derive adaptive meaning.

For example, when individuals attribute the cause of their stressful experience to themselves, they may focus on their own failures and feel regret and guilt (Roese et al., 2009; Joseph et al., 2012). These affects are associated with depression, PTSD, and intrusion (Arata and Burkhart, 1996; Roese et al., 2009). Additionally, because self-esteem and self-value are threatened when individuals fail, make a mistake, or act immorally, these behaviors are in contradiction with their global meaning, including self-worth (Janoff-Bulman, 1989). This results in a discrepancy between global and situational meanings. Hence, regret and guilt may promote threat evaluation and intrusive rumination.

However, self-blame and regret also have an adaptive function. In the case of self-blame with regards to own behavior during a stressful experience, most individuals consider whether they could have done something differently to have prevented the event. This process is known as counterfactual thinking (Davis et al., 1996). Behavioral self-blame is associated with perceived controllability of similar future events (Karl et al., 2009), as individuals are able to identify out what to do to avoid them. Thus, regret can help to improve performance. Regret also signals a need for corrective actions and leads individuals to implement them (Roese et al., 2009). Consequently, because individuals who feel regret and guilt become motivated to find meaning and actively attempt to use of their experience to improve behavior, regret and guilt may possibly promote deliberate rumination.

On the other hand, some events caused by others, such as betrayal, insult, and intentional infringement may cause anger (Nezlek et al., 2008). Anger is related to re-experience and intrusive memories of the traumatic event, which are two of the symptoms of PTSD (Kleim et al., 2013; Dewey et al., 2014). Given that these are similar concepts (Halligan et al., 2003) with some features (i.e., repetitive and difficult to control), in common with intrusive memories, thoughts, and rumination (Martin and Tesser, 1996), anger may promote intrusive rumination.

Some stressful experiences that are perceived to be caused by certain external objects; on the other hand, other experiences such as natural disasters, and unintentional injuries and accidents that are difficult to associate with specific objects. Since because these stressful events are not caused by others, individuals may find it difficult to control and deal with them to avoid reoccurrence. Furthermore, whatever the cause, individuals sometimes encounter situations that are hard to predict, control, or deal with, which seem to induce helplessness, fear (Tolstikova et al., 2005; O’Donnell et al., 2007), and sadness (Kitamura, 2006).

In summary, stressful and traumatic events that are unpredictable and uncontrollable may induce anger, helplessness, fear, and sadness. The stronger these negative affects are, the greater discrepancy there is between global and situational meanings. This could be because of the tendency of these events to disrupt individuals’ global meaning, which involves the belief that the world is predictable, comprehensible, and controllable (Janoff-Bulman, 1989). Moreover, because it is difficult to interpret and understand these stressful experiences in the framework of previous global meaning (as it is disrupted by such experiences) negative affect may induce threat evaluation and intrusive rumination.

### Purpose and Hypotheses of the Current Study

The purpose of current study was to examine the meaning-making processes with a focus on intrusive rumination, deliberate rumination, and negative affect. Specifically, we used structural equation modeling (SEM) to examine the relationships between threat evaluation, negative affect, two types of ruminations, and finding meaning in the stressful experience. The study hypotheses based on above discussion were as follows: (1) negative affect will be positively related to threat evaluation and intrusive rumination; (2) regret and guilt will be positively related to deliberate rumination; (3) threat evaluation will be positively related to both intrusive and deliberate rumination; (4) intrusive rumination immediately after the event will be positively related or not related to finding meaning; (5) deliberate rumination immediately after the event and at present will be positively related to finding meaning; (6) deliberate rumination immediately after the event will positively related to intrusive rumination at present; and (7) intrusive rumination at present will be negatively related to finding meaning.

## Materials and Methods

### Participants

This study carried out an investigation for undergraduate students. To prevent from being noticed the intention of this study, we recruited participants from some classes except the psychology. A total of 383 Japanese undergraduate students (200 male, 173 female, and 10 unknown) at a university in Japan in June 2015 participated in this study. The mean age of participants was 19.59 years (*SD* = 2.50, range = 18–30). They participated in this investigation during class.

### Procedure

First, participants were asked to remember the most stressful life event that they faced more than 1 year ago and describe it in writing in a blank section in the questionnaire. To avoid discomfort that may be brought about by recalling the stressful event, this study set the following conditions: (1) the event must have occurred more than 1 year ago, and (2) it had to be possible for participants to deeply reflect about the event. Second, participants completed the questionnaire which was comprised of the following questions regarding the event written to the questionnaire. Prior to the investigation, they were informed about the purpose of the study, that they did not need to answer any questions that made them uncomfortable, and that their personal information and data would be treated with strict confidentiality. Completion of the questionnaire took approximately 15 min. This study was approved by the institutional review board of the university.

### Measures

#### Demographic Information

Demographic data such as age and gender were self-reported.

#### Negative Affect

As for negative affect related to the stressful event, participants rated how they felt immediately after the stressful event in term of the following six negative affects: sadness, anger, regret, guilt, fear, and hopelessness, on a scale ranging from 1 (not at all) to 7 (strongly agree). These six items were selected based on the studies of Ellsworth and Smith (1988) and Scherer (2005). We used the following question: “When you experienced the event, to what extent did you feel each of the following emotions?”.

#### Threat Evaluation

To measure the degree of subjective evaluation of threat in relation to the event, we used the following single-item measure: “When you experienced the event, how much did you feel threatened by it?” Participants answered this item on a scale ranging from 0 (the least threatened in my life) to 100 (the most threatened in my life), which was based on Kamijo and Yukawa (2014, 2016).

#### Event Related Rumination Inventory

The Japanese version of the Event Related Rumination Inventory (Cann et al., 2011; Taku et al., 2015) is a 20-item inventory, with 10 items assessing intrusive, unintentional, and undesired thoughts and images, (i.e., intrusive rumination; e.g., “Thoughts about the event came to my mind and I could not stop thinking about them”), and the remaining 10 items assessing deliberate, more constructive, and purposeful thinking (i.e., deliberate rumination; e.g., “I thought about whether I have learned anything as a result of my experience”). Items were rated to a scale ranging from 1 (not at all) to 4 (often). Participants were asked to rate each item on how much they ruminated about the event at two points in time (i.e., immediately after the event and at present). In this study, we used only the three items with the highest factor loadings on each scale (Kamijo et al., 2016) in order to reduce participants’ burden to answer numerous items. The items of intrusive rumination were as follows: “Thoughts about the event came to mind and I could not stop thinking about them,” “I could not keep images or thoughts about the event from entering my mind,” and “Thoughts about the event distracted me or kept me from being able to concentrate.” The items of deliberate rumination were as follows: “I thought about whether I have learned anything as a result of my experience,” “I thought about whether I could find meaning from my life,” and “I thought about whether changes in my life have come from dealing with my experience.” The three items scores were, respectively, summed and averaged out to obtain the scores of intrusive and deliberate rumination. The internal consistencies for the scale were 0.90 (intrusive rumination immediately after the event), 0.96 (intrusive rumination at present), 0.84 (deliberate rumination immediately after the event), and 0.95 (deliberate rumination at present).

#### Finding Meaning

To measure whether participants found their own meaning in the stressful life event, they were asked the following question: “How much do you feel you have been able to make sense out of the event or find some kind of meaning in it?” They rated their answer on a scale from 1 (none) to 5 (a great deal). This statement was used to measure finding meaning in previous research (Davis et al., 1998; Updegraff et al., 2008; Kernan and Lepore, 2009; Park et al., 2012).

### Statistical Analysis

First, we described the study variables in terms of means and standard deviations. Then, we classified the stressful life events into five categories based on Taku et al. (2007). Associations between variables were measured using bivariate Pearson correlations.

Second, to examine the meaning-making processes focusing on negative affect and the two types of rumination, we used SEM based on the study hypotheses model. SEM can be viewed as a complex path model. The full information maximum likelihood estimation was used to generate the standardized parameter estimates. Because fit indexes represent different facets of model fit, we used multiple indexes: χ^2^ test, the comparative fit index (CFI), standardized root mean square residual (SRMR), and root-mean-square error of approximation (RMSEA). According to West et al. (2012), if CFI is over 0.95, RMSEA under 0.05, and SRMR under 0.10, the model is considered to have a good fit.

## Results

### Descriptive Statistics

Forty-one participants were excluded from the analysis due to the following reasons: they reported a stressful event that occurred within 1 year, they did not provide answers for more than half of all items, or their age was over 3 SDs from the average age (19.59 years). The final sample was 342 (183 male, 157 female, and 2 unknown; average age = 19.49 years, *SD* = 1.26). Descriptive data for all variables are presented in Table 1. We conducted an independent-samples *t*-test comparing all variables by gender, which showed no significant statistical difference between the genders for any variables except for fear [male average = 3.48 ± 2.09, female average = 4.40 ± 2.14, *t*(338) = 4.00, *p* < 0.001]. Hence, the following analyses did not assess gender differences.

**Table 1:** Means and standard deviations and correlations of all variables.

		*M*	*SD*	2	3	4	5	6	7	8	9	10	11	12
(1)	Threat evaluation	3.12	0.89	0.23^∗∗^	0.17^∗∗^	0.15^∗∗^	0.44^∗∗^	0.48^∗∗^	0.26^∗∗^	0.44^∗∗^	0.15^∗∗^	0.27^∗∗^	0.21^∗∗^	0.11^∗∗^
(2)	Regret	4.62	2.15	–	0.50^∗∗^	−0.08	0.37^∗∗^	0.13^∗^	0.39^∗∗^	0.32^∗∗^	0.30^∗∗^	0.21^∗∗^	0.18^∗∗^	0.19^∗∗^
(3)	Guilt	3.34	2.21		–	−0.12^∗^	0.23^∗∗^	0.21^∗∗^	0.25^∗∗^	0.25^∗∗^	0.22^∗∗^	0.15^∗∗^	0.11 ^∗^	0.10
(4)	Anger	4.60	1.95			–	0.09	0.00	0.11^∗^	0.09	−0.03	−0.02	−0.05	−0.06
(5)	Hopelessness	4.42	1.90				–	0.31^∗∗^	0.33^∗∗^	0.35^∗∗^	0.19^∗∗^	0.18^∗∗^	0.19^∗∗^	0.20^∗∗^
(6)	Fear	3.92	2.16					–	0.11	0.23^∗∗^	0.06	0.13^∗^	0.08	−0.01
(7)	Sadness	5.14	1.91						–	0.37^∗∗^	0.18^∗∗^	0.25^∗∗^	0.14^∗∗^	0.17^∗∗^
(8)	Intrusive rumination (immediately after the event)	2.90	0.86							–	0.24^∗∗^	0.36^∗∗^	0.26^∗∗^	0.25^∗∗^
(9)	Deliberate rumination (immediately after the event)	2.59	0.87								–	0.21^∗∗^	0.41^∗∗^	0.42^∗∗^
(10)	Intrusive rumination (at present)	1.58	0.86									–	0.57^∗∗^	0.13^∗^
(11)	Deliberate rumination (at present)	1.85	0.93										–	0.36^∗∗^
(12)	Finding meaning	3.02	1.40											–

The stressful events reported by participants in the current study were classified based on Taku et al. (2007): “self” (33.2%) included events such as a severe illness or injury, natural disaster, and any accident from club activities; “relationship” (28.5%) included events like being physically and/or verbally bullied at school, falling out with friend or teacher, and a relationship rupture; “school” (17.8%) included events such as failure on college entrance examination or any significant academic problem; “family” (9.6%) included events like parents’ divorce or separation, being abused by family member, and a family member’s illness; “bereavement” (6.0%) included events such as a death of a family member or loved one; “other” (4.9%) included events that did not fit into any of the five categories above. The time elapsed from the stressful event ranged from 1 to 15 years, with a mean of 3.75 (*SD* = 2.95) years.

### Path Analysis

Table 1 presents the correlation matrix for all variables. SEM was used to evaluate the path model, based on the study’s hypotheses: (a) in correspondence with Hypothesis 1, direct paths from all negative affects to threat evaluation and intrusive rumination immediately after the event; (b) in correspondence with Hypothesis 2, direct paths from regret and guilt to deliberate rumination immediately after the event; (c) in correspondence with Hypothesis 3, direct paths from threat evaluation to intrusive rumination immediately after the event and at present and deliberate rumination immediately after the event and at present; in correspondence with Hypotheses 4–7, (d) direct paths from intrusive (and deliberate) rumination immediately after the event to intrusive (and deliberate) rumination at present and finding meaning; (e) direct paths from intrusive (and deliberate) rumination at present to finding meaning; (f) correlations between intrusive rumination and deliberate rumination; (g) correlations among all negative affects. Furthermore, we aimed to examine the relationships between all negative affects and deliberate rumination: (h) direct paths from anger, sadness, hopelessness, and fear to deliberate rumination immediately after the event. Although it is possible that there is a relationship between finding meaning and threat evaluation, this link was not examined in this study. Taku et al. (2009, 2015) pointed out the importance of mental suffering, that is, rumination, for meaning making and PTG; thus, threat evaluation alone would not be sufficient to achieve this. As such, we considered the absence of a direct relationship between threat evaluation and finding meaning. Additionally, to exclude the effect of elapsed time, we included the direct paths from elapsed time to all variables.

**FIGURE 1:**
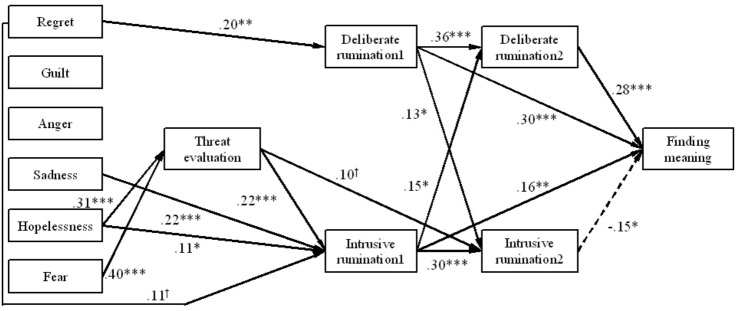
Path analysis of meaning making process. ^∗∗∗^*p* < 0.001, ^∗∗^*p* < 0.01, ^∗^*p* < 0.05, ^†^*p* < 0.10. The straight lines represent significant positive paths, long dashed lines represent significant negative paths. Intrusive (Deliberate) rumination1 = Intrusive (Deliberate) rumination immediately after the event, Intrusive (Deliberate) rumination2 = Intrusive (Deliberate) rumination at present.

Figure 1 presents the tested paths in the hypothesis model, which showed a good fit with the study data (χ^2^(31) = 65.197 (*p* < 0.001), CFI = 0.964, RMSEA = 0.057 [90%confidence interval = 0.037–0.076], SRMR = 0.034). Hopelessness and fear were positively associated with threat evaluation (hopelessness: β = 0.31, *p* < 0.001, fear: β = 0.40, *p* < 0.001). On the other hand, threat evaluation, hopelessness, sadness, and regret were positively associated with intrusive rumination immediately after the event (hopelessness: β = 0.11, *p* < 0.05, sadness: β = 0.22, *p* < 0.001, regret: β = 0.11, *p* < 0.10); however, only regret was correlated with deliberate rumination immediately after the event (β = 0.20, *p* < 0.01). Furthermore, finding meaning was positively associated with deliberate rumination, both immediately after the event and at present (immediately: β = 0.30, *p* < 0.001, present: β = 0.28, *p* < 0.001), as well as with intrusive rumination immediately after the event (β = -0.15, *p* < 0.01). Only intrusive rumination, however, was negatively associated with finding meaning (β = 0.16, *p* < 0.01). Additionally, intrusive rumination immediately after the event and deliberate rumination at present (β = 0.15, *p* < 0.05), as well as deliberate rumination immediately after the event and intrusive rumination at present (β = 0.13, *p* < 0.01) had positive correlations, respectively.

## Discussion

This is the first study to examine the relationships between the two types of rumination and meaning making, and the characteristics of negative affect in meaning making in a Japanese sample. In path analysis via SEM, Hypotheses 1, 2, and 4 were partially supported and Hypothesis 3, 5, 6, and 7 were mostly supported. Hence, individuals going through a stressful life event may experience a variety of negative affects and some find meaning in the event through rumination.

Although Hypotheses 1 and 2 were confirmed only partially, the results of the current study support the role of negative affect. First, regret was positively associated with both deliberate and intrusive rumination. Comparing the ideal world and the situation caused by themselves, individuals often regret their actions. Roese et al. (2009) identified repetitive regret, which involves regrets repeatedly coming to mind, such as focusing on self-blame and “what-might-have-been” thoughts. This view is in agreement with the results of this study regarding the association between regret and intrusive rumination. Additionally, regret induced deliberate rumination. Regret can help improve performance, as it signals the need for corrective actions and pushes individuals into implementing them (Roese et al., 2009). Hence, those who regret their actions resulting in a stressful event may somehow be motivated to make the best use of the experience, attempt to prevent similar events, and be able to deal with them successfully.

On the other hand, unlike regret, guilt was not correlated with any variables in the current study. Guilt is a self-conscious affect that relates to a sense of responsibility in the cause of harm to others (Lee et al., 2001; Berndsen et al., 2004). When individuals experience a stressful event, they often seem to blame themselves more than others, as this enables them to maintain their pre-existing global meaning, or at least minimizes the need for it to change (Janoff-Bulman, 1989). As such, by recognizing their own fault soon, it is easier for them to understand and interpret their experiences within the framework of their pre-existing global meaning. Since they can protect their beliefs and worldviews, they do not need to reconstruct or repair their global meaning, which will likely remain intact.

Moreover, helplessness, sadness, and fear were positively associated with threat evaluation and intrusive rumination, which reflects the degree of distress and discrepancy between global and situational meanings. These affects are likely to occur when individuals experience stressful events that are difficult to predict, control, and deal with. If individuals believe that they have little control over life events, they are more likely to feel hopeless, frightened, and sad (Tolstikova et al., 2005; Kitamura, 2006; O’Donnell et al., 2007; Reiland et al., 2014). These negative affects may disrupt global meaning (e.g., “Our world is predictable and safety” and “We can directly control our world through our own behaviors”); thus, the discrepancy between global and situational meanings may increase. Furthermore, because it is difficult to interpret and understand their experience based on the framework of previous global meaning, negative affect and discrepancy may induce intrusive rumination. That is, when individuals experience stressful events that cannot be controlled and dealt with by themselves, they may experience hopelessness, fear, and sadness. When this happens, their global meaning is likely to be disrupted, and intrusive rumination may increase because of the greater discrepancies.

In contrast, anger was not associated with any variables in the current study, although we had predicted a positive relationship between anger and intrusive rumination. This may be because the current study employed a retrospective method, i.e., recalling past stressful and traumatic experiences and answering questions about them. Generally, in prior studies, anger and rumination were positively related (Speckens et al., 2007; Kleim et al., 2013; Dewey et al., 2014; White and Turner, 2014). However, some research reported the adaptive function of anger, which was associated with PTG and induced adaptive coping such as active effort actions (Park et al., 2008, 2012). Hence, although anger is often correlated with rumination (Watkins, 2008; Kleim et al., 2013; Dewey et al., 2014), individuals who feel anger may, however, tend to engage in active coping actions. Consequently, this active coping action after the stressful event may reconstruct the memory that is accompanied by anger; thus, the association between anger and intrusive rumination cannot be reflected in a study using a retrospective method. Further research is thereby recommended.

Furthermore, the current study revealed that the effects of negative affect varied according to the kind of affect. Namely, some negative affects such as helplessness, sadness, and fear may disrupt individuals’ global meaning and induce rumination that is intrusive, uncontrollable, and unwanted, which could become an indirect factor interfering with finding meaning. In contrast, other negative affects such as regret, guilt, and anger may not always be maladaptive in relation to meaning making. Specifically, regret may promote deliberate rumination as revealed in this study, and can signal a need for corrective actions and lead individuals to implement them (Roese et al., 2009).

The results showed that threat evaluation induced intrusive rumination but not deliberate rumination; thus, Hypothesis 3 was not completely supported. Discrepancy between global and situational meanings leads to uncontrollable and unwanted images and thoughts, which indicates that individuals have not yet successfully processed their experiences (Joseph and Williams, 2005). This, in effect, signals the need to find meaning to reduce the discrepancy (Helgeson et al., 2006). Given that there is a positive correlation between intrusive and deliberate rumination, intrusive rumination can generate further intentional cognitive processes such as deliberate rumination. Future research is needed to clarify their relationship.

As predicted, deliberate rumination both immediately after stressful experiences and at present promoted finding meaning, while intrusive rumination at present inhibited it. These results supported Hypotheses 4, 5, 6, and 7. Deliberate rumination involves perceiving multilateral sides of the stressful experience including value, meaning, and significance (Calhoun et al., 2000; Cann et al., 2011), and may also decrease the discrepancy between global and situational meanings, as it promote finding meaning. Furthermore, when intrusive rumination still occurs frequently a long time after the stressful event, this may indicate that the discrepancy has not yet decreased, which may interfere with finding constructive meaning, as individuals are likely to pay attention to negative information, images, and thoughts regarding the stressful experience and cannot disengage from it (Koster et al., 2011; Whitmer and Gotlib, 2013).

Additionally, partially supporting Hypothesis 4, intrusive rumination immediately after the stressful events was also related to deliberate rumination at present and finding meaning. As mentioned in prior studies, intrusive rumination is a trigger for the cognitive processes toward deliberate rumination and positive change (Taku et al., 2009) and leads to rich memory of the experience (Krans et al., 2009). According to Joseph et al. (2012), intrusive rumination immediately after stressful events is a normal reaction in response to the traumatic experience. This evidence supports the results of this study, that is, intrusive rumination immediately after stressful events is surely a factor of distress; however, it does not necessarily lead to maladaptation or poor mental health.

Aside from the positive effects of deliberate rumination immediately after a stressful event on finding meaning, there was also a positive effect of this type of rumination on intrusive rumination at present, as described in Hypothesis 6. Hence, it is possible that deliberate rumination may also induce distress and psychological problems. Deliberate engagement in meaning making against one’s will may not lead to adaptive meaning making, but contribute to later psychological stress and enhancement of intrusive rumination (Folkman, 2008; Nightingale et al., 2010; Kamijo and Yukawa, 2016). Therefore, it is assumed that the appropriate timing of the two types of rumination for adaptive meaning making may be different.

Finally, several limitations of the present study should be noted. First, due to the cross-sectional nature of our dataset, true mediation could not be established because of a lack of temporal ordering. Additionally, we examined the relationship between negative affect and the two types of rumination that follow immediately after the event, but could not establish a precise mutual relationship. Given that there may be an interaction between them (Joseph et al., 2012), future research needs to implement a longitudinal investigation to reveal the change process of meaning making based on rumination.

Second, there is a possibility of recollection bias in the retrospective method that we used. When negative affect experienced immediately after stressful events is assessed retrospectively, memory and reporting biases can occur (Nightingale et al., 2010; Bonanno, 2013). Moreover, it is difficult to memorize an experience exactly (Park, 2010), and those who found meaning may reconstitute their memory to ensure consistency with their current interpretation and evaluation of the stressful experience (Bluck, 2003; Dekel and Bonanno, 2011). Consequently, future research needs to use other methods beyond retrospective data collection, such as longitudinal investigation or diary method.

Third, we did not consider the contents of the meaning found by participants. Not all meanings are necessarily positive. Even if individuals can find meaning, if this involves negative beliefs, worldview, and self-concept, and is accompanied by a feeling of disgust, it may lead to aggravation of distress, depression, and PTSD (Joseph and Linley, 2005; Payne et al., 2007; Park, 2008; Joseph, 2009).

Finally, in light of the results of this study, more research is needed on the various dimensions and types of meaning (Park, 2010). More importantly, future research should pay attention to not only the degree of finding meaning but also the contents of such meaning. It is through knowledge that we will be able to understand how meaning making could be a central and integral part of life.

## Ethics Statement

This study was carried out in accordance with the recommendations of research ethics committee in University of Tsukuba. The protocol was approved by there. All subjects gave written informed consent in accordance with the Declaration of Helsinki.

## Author Contributions

NK and SY has made an important contribution in writing this paper. NK took the responsibility as first author and led the writing work.

## Conflict of Interest Statement

The authors declare that the research was conducted in the absence of any commercial or financial relationships that could be construed as a potential conflict of interest.

## References

[B1] Arata C. Burkhart B. (1996). Post-traumatic stress disorder among college victims of acquaintance assault. *J. Psychol. Hum. Sex.* 8 79–92. 10.1300/J056v08n01_06 21526600

[B2] Berndsen M. van der Pligt J. Doosje B. Manstead A. S. R. (2004). Guilt and regret: the determining role of interpersonal and intrapersonal harm. *Cognit. Emot.* 18 55–70. 10.1080/02699930244000435

[B3] Bluck S. (2003). Autobiographical memory: exploring its functions in everyday life. *Memory* 11 113–123. 10.1080/741938206 12820825

[B4] Bonanno G. A. (2013). Meaning making, adversity, and regulatory flexibility. *Memory* 21 150–156. 10.1080/09658211.2012.745572 23311413 PMC3565080

[B5] Bower J. E. Kemeny M. E. Taylor S. E. Fahey J. L. (1998). Coping processing, discovery of meaning CD4 decline, and AIDS-related mortality among bereaved HIV-seropositive men. *J. Consult. Clin. Psychol.* 66 979–986. 10.1037/0022-006X.66.6.979 9874911

[B6] Calhoun L. G. Cann A. Tedeschi R. G. McMillan J. (2000). A correlational test of the relationship between posttraumatic growth, religion, and cognitive processing. *J. Trauma. Stress* 13 521–527. 10.1023/A:1007745627077 10948491

[B7] Cann A. Calhoun L. G. Tedeschi R. G. Triplett K. N. Vishnevsky T. Lindstrom C. M. (2011). Assessing posttraumatic cognitive processes: the event related rumination inventory. *Anxiety Stress Coping* 24 137–156. 10.1080/10615806.2010.529901 21082446

[B8] Davis C. G. Lehman D. R. Silver R. C. Wortman C. B. Ellard J. H. (1996). Self-blame following a traumatic event: the role of perceived avoidability. *Pers. Soc. Psychol. Bull.* 22 557–567. 10.1177/0146167296226002

[B9] Davis C. G. Macdonald S. L. (2004). Threat appraisals, distress and the development of positive life changes after September 11th in a Canadian sample. *Cognit. Behav. Ther.* 33 68–78. 10.1080/16506070410025832 15279312

[B10] Davis C. G. Nolen-Hoeksema S. Larson J. (1998). Meaning sense of loss and benefiting from the experience: two construal of meaning. *J. Pers. Soc. Psychol.* 75 561–574. 10.1037/0022-3514.75.2.561 9731325

[B11] Davis C. G. Novoa D. C. (2013). Meaning-making following spinal cord injury: individual difference and within-person change. *Rehabil. Psychol.* 58 166–177. 10.1037/a0031554 23437993

[B12] Dekel S. Bonanno G. A. (2011). Changes in trauma memory and patterns of posttraumatic stress. *Psychol. Trauma* 5 26–34. 10.1037/a0022750

[B13] Dewey D. Schuldberg D. Madathil R. (2014). Do peritraumatic emotions differentially predict PTSD symptom clusters? Initial evidence for emotion specificity. *Psychol. Rep.* 115 1–12. 10.2466/16.02.PR0.115c11z7 25153945

[B14] Ellsworth P. C. Smith C. A. (1988). From appraisal to emotion: differences among unpleasant feelings. *Motiv. Emot.* 12 271–302. 10.1007/BF00993115

[B15] Folkman S. (2008). The case for positive emotions in the stress process. *Anxiety Stress Coping* 21 3–14. 10.1080/10615800701740457 18027121

[B16] George L. S. Park C. L. (2013). Are meaning and purpose distinct? An examination of correlates and predictors. *J. Positive Psychol.* 8 365–375. 10.1080/17439760.2013.805801

[B17] Gillies J. Neimeyer R. A. (2006). Loss, grief, and the search for significance: toward a model of meaning reconstruction in bereavement. *J. Constr. Psychol.* 19 31–65. 10.1080/10720530500311182

[B18] Greenberg M. A. (1995). Cognitive processing of traumas: the role of intrusive thoughts and reappraisals. *J. Appl. Soc. Psychol.* 25 1262–1296. 10.1111/j.1559-1816.1995.tb02618.x

[B19] Halligan S. L. Michael T. Clark D. M. Ehlers A. (2003). Posttraumatic stress disorder following assault: the role of cognitive processing, traumamemory, and appraisal. *J. Consult. Clin. Psychol.* 71 419–431. 10.1037/0022-006X.71.3.419 12795567

[B20] Helgeson V. S. Reynolds K. A. Tomich P. L. (2006). A meta-analytic review of benefit finding and growth. *J. Consult. Clin. Psychol.* 74 797–816. 10.1037/0022-006X.74.5.797 17032085

[B21] Janoff-Bulman R. (1989). Assumptive worlds and the stress of traumatic events: applications of the schema construct. *Soc. Cognit.* 7 113–136. 10.1521/soco.1989.7.2.113

[B22] Joseph S. (2009). Growth following adversity: positive psychological perspectives on posttraumatic stress. *Psychol. Topics* 18 335–344.

[B23] Joseph S. Linley P. A. (2005). Positive adjustment to threatening events: an organismic valuing theory of growth through adversity. *Rev. Gen. Psychol.* 9 262–280. 10.1037/1089-2680.9.3.262

[B24] Joseph S. Murphy D. Regal S. (2012). An affective-cognitive processing model of post-traumatic growth. *Clin. Psychol. Psychother.* 19 316–325. 10.1002/cpp.1798 22610981

[B25] Joseph S. Williams R. (2005). Understanding posttraumatic stress: theory, reflection, context and future. *Behav. Cognit. Psychother.* 33 423–441. 10.1017/S1352465805002328

[B26] Kamijo N. Taku K. Yukawa S. (2016). Gender differences in the role of intrusive and deliberate ruminations on posttraumatic growth after 3.11. *Paper Presented at the 31st International Congress of Psychology 2016*, Yokohama.

[B27] Kamijo N. Yukawa S. (2014). Examination of rumination and meaning making in stressful events: the influence of subjective evaluation of events and personal characteristics. *Jpn. J. Psychol.* 85 445–454. 10.4992/jjpsy.85.13047 25639027

[B28] Kamijo N. Yukawa S. (2016). The role of intrusive and deliberate ruminations for meaning making in stressful events. *Jpn. J. Psychol.* 86 513–523. 10.4992/jjpsy.86.14037 26964366

[B29] Karl A. Rabe S. Zllner T. Maercker A. Stopa L. (2009). Negative self-appraisals in treatment-seeking survivors of motor vehicle accidents. *J. Anxiety Disord.* 23 775–781. 10.1016/j.janxdis.2009.03.001 19369030

[B30] Kernan W. D. Lepore S. J. (2009). Searching for and making meaning after breast cancer: prevalence, patterns, and negative affect. *Soc. Sci. Med.* 68 1176–1182. 10.1016/j.socscimed.2008.12.038 19157667

[B31] Kitamura H. (2006). “Kanjo no aratana igi,” in *Kanjo Kenkyu no Shintenkai*, eds Kitamura H. Kimura H. (Japan: Nakanishiya Shuppan), 3–19.

[B32] Kleim B. Graham B. Bryant R. A. Ehlers A. (2013). Capturing intrusive re-experiencing in trauma survivors’ daily lives using ecological momentary assessment. *Memory* 122 998–1009. 10.1037/a0034957 24364602 PMC3906879

[B33] Koster E. H. W. Lissnyder E. D. Derakshan N. Raedt R. D. (2011). Understanding depressive rumination from a cognitive science perspective: the impaired disengagement hypothesis. *Clin. Psychol. Rev.* 31 138–145. 10.1016/j.cpr.2010.08.005 20817334

[B34] Krans J. Näring G. Becker E. S. Holmes E. A. (2009). Intrusive trauma memory: a review and functional analysis. *Appl. Cognit. Psychol.* 23 1076–1088. 10.1002/acp.1611

[B35] Lee D. A. Scragg P. Turner S. (2001). The role of shame and guilt in traumatic events: a clinical model of shame-based and guilt-based PTSD. *Br. J. Med. Psychol.* 74 451–466. 10.1348/000711201161109 11780793

[B36] Martin L. L. Tesser A. (1996). “Some ruminative thoughts,” in *Ruminative thoughts: Advances in Social Cognition* Vol. 9 ed. Wyer R. S. Jr. (Mahwah, NJ: Lawrence Erlbaum Associates), 1–47.

[B37] Michael T. Halligan S. L. Clark D. M. Ehlers A. (2007). Rumination in posttraumatic stress disorder. *Depress. Anxiety* 24 307–317. 10.1002/da.20228 17041914

[B38] Nezlek J. B. Vansteelandt K. Mechelen I. V. Kuppens P. (2008). Appraisal-emotion relationships in daily life. *Emotion* 8 145–150. 10.1037/1528-3542.8.1.145 18266526

[B39] Nightingale V. R. Sher T. G. Hansen N. B. (2010). The impact of receiving an HIV diagnosis and cognitive processing on psychological distress and posttraumatic growth. *J. Trauma Stress* 23 452–460. 10.1002/jts.20554 20648562 PMC3629914

[B40] O’Donnell M. L. Elliott P. Wolfgang B. J. Creamer M. (2007). Posttraumatic appraisals in the development and persistence of posttraumatic stress symptoms. *J. Trauma. Stress* 20 173–182. 10.1002/jts.20198 17427908

[B41] Park C. L. (2008). Testing the meaning making model of coping with loss. *J. Soc. Clin. Psychol.* 27 970–994. 10.1521/jscp.2008.27.9.970 28164236

[B42] Park C. L. (2010). Making sense of the meaning literature: an integrative review of meaning making and its effects on adjustment to stressful life events. *Psychol. Bull.* 136 257–301. 10.1037/a0018301 20192563

[B43] Park C. L. Aldwin C. M. Fenster J. R. Snyder L. B. (2008). Pathways to posttraumatic growth versus posttraumatic stress: coping and emotional reactions following the September 11,2001, terrorist attacks. *Am. J. Orthopsychiatry* 78 300–312. 10.1037/a0014054 19123749

[B44] Park C. L. George L. S. (2013). Assessing meaning and meaning making in the context of stressful life events: measurement tools and approaches. *J. Posit. Psychol.* 8 483–504. 10.1080/17439760.2013.830762

[B45] Park C. L. Riley K. E. Snyder L. B. (2012). Meaning making coping, making sense, and post-traumatic growth following the 9/11 terrorist attacks. *J. Posit. Psychol.* 7 198–207. 10.1080/17439760.2012.671347

[B46] Payne A. J. Joseph S. Tudway J. (2007). Assimilation and processes following traumatic experiences. *J. Loss Trauma* 12 75–91. 10.1080/15325020600788206

[B47] Reiland S. A. Lauterbach D. Harrington E. F. Palmieri P. A. (2014). Relationships among dispositional attributional style, trauma-specific attributions, and PTSD symptoms. *J. Aggress. Maltreat. Trauma* 23 823–841. 10.1080/10926771.2014.941083

[B48] Roberts K. J. Lepore S. J. Helgeson V. S. (2006). Social-cognitive correlated of adjustment to prostate cancer. *PsychoOncology* 15 183–192. 10.1002/pon.934 15929030 PMC2610315

[B49] Roese N. J. Epstude K. Fessel F. Morrison M. Smallman R. Summerville A. (2009). Repetitive regret, depression, and anxiety: findings from nationally representative survey. *J. Soc. Clin. Psychol.* 28 671–688. 10.1521/jscp.2009.28.6.671

[B50] Scherer K. R. (2005). What are emotions? And how can they be measured? *Soc. Sci. Inf.* 44 695–729. 10.1177/0539018405058216

[B51] Silver R. L. Boon C. Stones M. H. (1983). Searching for meaning in misfortune: making sense of incest. *J. Soc. Issues* 39 81–102. 10.1111/j.1540-4560.1983.tb00142.x

[B52] Speckens A. E. M. Ehlers A. Hackmann A. Ruths F. A. Clark D. M. (2007). Intrusive memories and rumination in patients with post-traumatic stress disorder: a phenomenological comparison. *Memory* 15 249–257. 10.1080/09658210701256449 17454662

[B53] Taku K. Calhoun L. G. Cann A. Tedeschi R. G. (2008). The role of rumination in the coexistence of distress and posttraumatic growth among bereaved Japanese university students. *Death Stud.* 32 428–444. 10.1080/07481180801974745 18767236

[B54] Taku K. Calhoun L. G. Tedeschi R. G. Gil-Rivas V. Kilmer R. P. Cann A. (2007). Examining posttraumatic growth among Japanese university students. *Anxiety Stress Coping* 20 353–367. 10.1080/10615800701295007 17999236

[B55] Taku K. Cann A. Tedeschi R. G. Calhoun L. G. (2009). Intrusive versus deliberate rumination in posttraumatic growth across US and Japanese samples. *Anxiety Stress Coping* 22 129–136. 10.1080/10615800802317841 18937084

[B56] Taku K. Cann A. Tedeschi R. G. Calhoun L. G. (2015). Core beliefs shaken by an earthquake correlate with posttraumatic growth. *Psychol. Trauma* 7 563–569. 10.1037/tra0000054 26010110

[B57] Tedeschi R. G. Calhoun L. G. (2004). Posttraumatic growth: conceptual foundations and empirical evidence. *Psychol. Inq.* 15 1–18. 10.1207/s15327965pli1501_01

[B58] Tolstikova K. Fleming S. Chartier B. (2005). Grief, complicated grief, and trauma: the role of the search for meaning, impaired self-reference, and death anxiety. *Illness Crisis Loss* 13 293–313. 10.1177/105413730501300402

[B59] Updegraff J. A. Silver R. C. Holman E. A. (2008). Searching for and finding meaning in collective trauma: results from a national longitudinal study of the 9/11 terrorist attacks. *J. Pers. Soc. Psychol.* 95 709–722. 10.1037/0022-3514.95.3.709 18729704 PMC2617710

[B60] Watkins E. R. (2008). Constructive and un-constructive repetitive thought. *Psychol. Bull.* 134 163–206. 10.1037/0033-2909.134.2.163 18298268 PMC2672052

[B61] West S. G. Taylor A. B. Wu W. (2012). “Model fit and model selection in structural equation modeling,” in *Handbook of Structural Equation Modelling*, ed. Hoyle R. H. (New York, NY: Guilford Press), 209–231.

[B62] White B. A. Turner K. A. (2014). Anger rumination and effortful control: mediation effects on reactive but not proactive aggression. *Pers. Individ. Differ.* 56 186–189. 10.1016/j.paid.2013.08.012

[B63] Whitmer A. J. Gotlib I. H. (2013). An attentional scope model of rumination. *Psychol. Bull.* 139 1036–1061. 10.1037/a0030923 23244316 PMC3773498

[B64] Wu X. Zhou X. Wu Y. An Y. (2015). The role of rumination in posttraumatic stress disorder and posttraumatic growth among adolescents after the wenchuan earthquake. *Front. Psychol.* 6:1335. 10.3389/fpsyg.2015.01335 26388826 PMC4559646

